# Editorial: Impact of COVID-19 pandemic on eating habits: 2-year legacy of social distancing times

**DOI:** 10.3389/fnut.2023.1188630

**Published:** 2023-05-04

**Authors:** William R. Tebar, Danilo R. Silva, Diego G. D. Christofaro

**Affiliations:** ^1^Center for Clinical and Epidemiological Research, University Hospital, University of São Paulo, São Paulo, Brazil; ^2^Department of Physical Education, Federal University of Sergipe, São Cristóvão, Brazil; ^3^Department of Physical Education, São Paulo State University, São Paulo, Brazil

**Keywords:** eating habits, food choices, dietary pattern, COVID-19, pandemic, social distancing

This Research Topic addressed the 2-year legacy of social distancing times caused by COVID-19 pandemic on the eating habits from most diverse populations worldwide. The social and sanitary policies during pandemic substantially affected the schedules and lifestyle of many people, with longer periods staying at home and consequences on their eating habits and food choices (1).

Dhakal et al. observed a decline of 12.6% in food spending by Americans during the first year of pandemic, when compared to the previous 4 years. This study reported a decline of 33.7% in the expenses with food away from home and an increase of 6.9% in eating at home expenditure. Many people joined to delivery options for fast-foods consumption during pandemic, affecting the nutritional content of meals, which may not specifically reflect a healthier food adherence.

Otherwise, eating at home has been associated with healthy eating habits even before the pandemic, mainly considering how the food is prepared (2). Maharat et al. observed positive changes in the variety of food and beverages intake, but also a significant increase in body mass index of Iranian adults during pandemic. Furthermore, this study observed that consumption of more nutritious foods was associated with higher socioeconomic condition. The family income was substantially impacted by restrictions or commitment to certain work activities and job loss (3), aggravated by the rising prices of foods (4). The worry of food products being out of stock led people to compulsive purchases of food in larger amounts than usual, leading to an excessive availability of food at home and preference to stock less perishable products than fresh foods.

Unhealthy eating habits and worse mental health were also associated since before pandemic (5). The many worries, fears, and boredom during COVID-19 were trigger points to feelings of anxiety, depression, and stress (6). The study of Anampa-Canales et al. identified that for every four households, three had experienced some food insecurity during pandemic in Peru. This study reported that food insecurity was independently associated with worse mental health, where participants that experienced food insecurity were up to 10 times more likely to have moderate-to-severe anxiety, besides being more likely to have depression and stress.

Changes in eating habits of special populations were also covered by this Research Topic. Vaghef-Mehrabani et al. assessed 9,870 pregnant women during pandemic and observed that 54.3% of them reported changes on eating habits. Negative dietary changes were associated with non-White ethnicity, job loss, anxiety and depression symptoms, self-isolation, fear of COVID-19, and pandemic phase. The decrease in the consumption of fresh vegetables and fruits and the increase in the consumption of sweets and snacks were associated with worse mental health symptoms in this study. The healthy eating habits of pregnant women is substantially important to avoid pregnancy complications and adverse child health outcomes, precluding excessive gestational weight gain, and improving lactation (7).

People with diabetes are another special group affected by pandemic restrictions. A systematic review by Lashkarbolouk et al. reported that eating habits of diabetic people vary from country to country. This review observed an increase in snacks and sweets in some studies, leading to weight gain in the participants. However, other studies in this review observed an increase in the consumption of grains, fruits, and vegetables, as well as a decrease in consumption of alcoholic beverages, snack, and sweets during pandemic. The fear of COVID-19 consequences allied to the difficulty for medication access and for the routine glycemic monitoring may have led diabetic patients to consume healthier foods for management of their body weight and blood sugar. However, the excessive time at home, missing social activities and feeling of tiredness and boredom may have contributed to increase meals frequency and snacking, even when not hungry.

Several metabolic complications have been associated with weight gain, such as elevated plasma glucose, raised blood pressure, dyslipidemia, and inflammatory state (8). The study of Ko et al. reported no significant changes in anthropometric indicators and body fat during pandemic among Korean adults, but higher odds of metabolic syndrome were observed during social distancing period than the previous two annually visits, with a sharp increase in young adults. The decrease in food quality consumption and increased sedentary behavior during pandemic are factors that may have compromised metabolic health regardless prominent weight gain in this population.

The younger population was one of the most affected by pandemic, with a huge emotional and behavioral burden (9). The systematic review by Pourghazi et al. reported both positive and negative changes in the eating habits of children and adolescents during pandemic. Positive changes were related to a reduction in fast food and soft drink consumption, while a decrease in fruits and vegetables consumption and increase in snacking and consumption of sweets were also reported. The temporary closure of schools may have led the children to have more time to make regular meals and higher frequency of home-cooked foods consumption supervised by parents instead of eating at malls and restaurants. Otherwise, the increased time in TV watching and mobile screen devices led children and adolescents to higher snacking between meals and at night.

Another important special group affected by pandemic was the elderly, where healthy dietary habits have been associated with better health outcomes since before pandemic (10). Laskou et al. reported that 4.9% of men and 9.4% of women had poorer diet quality during pandemic when compared to before the first lockdown. It was also observed that the worse change in diet quality was associated with lower education, poor nutritional status, and higher sarcopenia score among older community-dwelling adults.

In general, changes in eating habits during pandemic seemed mixed and controversial, varying between and within countries, life stage and socioeconomic backgrounds. Therefore, this is time to surveillance and to intervene on eating habits both to maintain the positive changes as well as focusing on the reduction of negative changes observed during the COVID-19 pandemic, mainly caused by mental health worsening and sedentary lifestyle. This Research Topic indicates research directions and perspectives for public policies and clinical practice in the field of nutrition.

## Author contributions

All authors listed have made a substantial, direct, and intellectual contribution to the work and approved it for publication.
